# HEALTH SYSTEM LEADERSHIP PERSPECTIVES AND PRIORITIES ON CARE FOR OLDER ADULTS

**DOI:** 10.1093/geroni/igad104.3383

**Published:** 2023-12-21

**Authors:** Emily Franzosa, Lynette Kelley, Eileen Dryden, Lauren Moo, William Hung

**Affiliations:** Icahn School of Medicine at Mount Sinai, New York City, New York, United States; VA Eastern Colorado Health Care System, Aurora, Colorado, United States; VA Bedford Healthcare System, Bedford, Massachusetts, United States; VA Bedford Healthcare System, Bedford, Massachusetts, United States; Icahn School of Medicine at Mount Sinai, New York City, New York, United States

## Abstract

Innovative care models for older adults such as non-institutional and home-based care have the potential to improve quality and efficiency of healthcare, but adoption of these models requires resources from health systems. Health systems leaders can influence the relative priority of geriatric care and associated resource allocation. This influence is especially important for areas with limited resources such as in rural areas. We interviewed recently retired health system leaders about their experience and how priorities in health systems can be aligned to improve the quality and reach of geriatric care. We conducted qualitative interviews with 10 participants (40% female) using a snowball sampling strategy of geographically diverse, retired hospital, regional, and national leaders in the Veteran Healthcare system, the largest single-payer system in the US. Participants described how they managed myriad competing health system priorities by weighing and devoting attention and time to tackle the crises of the day, the numerous national mandates and directives, and the goals for the local hospital or system, as seen from their own perspectives. Alignment with performance metrics provides strong impetus for some health system leaders to determine their priorities. Incentives to encourage health systems leaders to prioritize and allocate resources aimed at long-term improvements in care could be important. Additionally, building relationships and maintaining trust amongst partners across facilities and offices is critical to initiating and growing geriatrics programs. This work highlights that influence must work on multiple levels to effect change in prioritization of care for older adults.

